# Are we failing to protect threatened mangroves in the Sundarbans world heritage ecosystem?

**DOI:** 10.1038/srep21234

**Published:** 2016-02-16

**Authors:** Swapan K. Sarker, Richard Reeve, Jill Thompson, Nirmal K. Paul, Jason Matthiopoulos

**Affiliations:** 1 Institute of Biodiversity, Animal Health and Comparative Medicine, University of Glasgow, Glasgow G12 8QQ, United Kingdom; 2 Centre for Ecology & Hydrology, Bush Estate, Penicuik, Midlothian, EH26 0QB, United Kingdom; 3 Management Plan Division, Bangladesh Forest Department, Khulna 9100, Bangladesh; 4 Department of Forestry and Environmental Science, Shahjalal University of Science & Technology, Bangladesh

## Abstract

The Sundarbans, the largest mangrove ecosystem in the world, is under threat from historical and future human exploitation and sea level rise. Limited scientific knowledge on the spatial ecology of the mangroves in this world heritage ecosystem has been a major impediment to conservation efforts. Here, for the first time, we report on habitat suitability analyses and spatial density maps for the four most prominent mangrove species - *Heritiera fomes, Excoecaria agallocha, Ceriops decandra* and *Xylocarpus mekongensis*. Globally endangered *H. fomes* abundances declined as salinity increased. Responses to nutrients, elevation, and stem density varied between species. *H. fomes* and *X. mekongensis* preferred upstream habitats. *E. agallocha* and *C. decandra* preferred down-stream and mid-stream habitats. Historical harvesting had negative influences on *H. fomes, C. decandra* and *X. mekongensis* abundances. The established protected area network does not support the most suitable habitats of these threatened species. We therefore recommend a reconfiguration of the network to include these suitable habitats and ensure their immediate protection. These novel habitat insights and spatial predictions can form the basis for future forest studies and spatial conservation planning, and have implications for more effective conservation of the Sundarbans mangroves and the many other species that rely on them.

The mangrove biome, spanning over 137,760 km^2^ of coastal areas in 118 countries is under severe threat. Nearly 50% of the biome has been lost since the 1950s because of inadequate habitat protection, and large-scale habitat alteration^1^. If the current rate of mangrove loss continues, the whole mangrove biome will disappear in the next 100 years^2^. There are only 70 mangrove species worldwide, compared to between 40,000 and 53,000 tropical forest tree species^3^. Already 16% of mangrove species are critically endangered, endangered or vulnerable and 10% are near-threatened^4^. More than 40% of the mangrove-endemic vertebrates are now also at risk of extinction due to habitat loss^5^.

The Sundarbans stretches along the coast of Bangladesh (6,017 km^2^) and India (4,000 km^2^) and forms the largest single block of halophytic mangrove forest in the world. This unique ecosystem provides the breeding and nursing habitats for diverse marine organisms, houses the last habitats of many endangered animals eg. Royal Bengal tiger (*Panthera tigris*) and Ganges river dolphin (*Platanista gangetica*), supports the livelihoods of about 3.5 million coastal dwellers and helps reduce the death toll of tsunamis and cyclones^6^ in the area. It was designated a Ramsar site under the Ramsar Convention in 1992^7^. UNESCO declared the Sundarbans a World Heritage Site in 1997, because of its ‘Outstanding Universal Value’, biological diversity and the ecosystem services the area provides^7^.

Historical human pressures (i.e. over-exploitation, dam construction, shrimp and salt farming, and regular oil spills) have severely degraded the Sundarbans ecosystem by depleting forest tree stock^8^. Sundarbans is a sea-dominated delta, where freshwater river flows help to modulate salt-water toxicity and keep the ecosystem suitable for mangrove trees. Ganges’ freshwater flow into the Sundarbans has dropped from 3700 m^3^ s^−1^ to 364 m^3^ s^−1^ since the construction of the Farakka dam in India^9^ in 1974. In addition, the rate of sea level rise (SLR) along the Bangladesh coast (5.93 mm yr^−1^) in the last century was substantially higher than the global average (1.0–2.0 mm yr^1^)^10^. The National Adaptation Program of Action has projected 32 cm and 88 cm of SLR by 2050 and 2100, respectively. The Sundarbans as an already stressed ecosystem is likely to be less resilient to the impact of climate change.

In Sundarbans, the population of the globally endangered species, *H. fomes*, is estimated to have declined by 76% since 1959 and about 70% of the remaining *H. fomes* trees are affected by the ‘top dying’ disease^11^. Dramatic declines in other dominant mangrove species (e.g. *E. agallocha* and *X. mekongensis*) have also been reported^12^. We have limited understanding of the current spatial distributions of *C. decandra*, a globally near-threatened species^7^. Future climate scenarios (in particular for SLR) and ongoing habitat degradation may alter the current spatial distributions of these mangrove species and forest community composition.

A limited understanding of mangrove spatial distributions and mangrove species habitat requirements has reduced the success of conservation initiatives in many countries^13^, including Bangladesh^14^. Only recently have coastal mangrove distributions been modelled at global^15^ and regional^16^ scales and we are now in urgent need of Habitat Suitability Models (HSMs), based on fine-scale species abundance and environmental data to assist us in protecting threatened ecosystems such as the Sundarbans. HSMs and their outputs (i.e. habitat maps) are widely used during different phases of resource management and spatial conservation planning^17^. These maps are also used to identify areas appropriate for establishing protected areas, evaluate threats to those areas, and design reserves^18^. For example, a baseline distribution map of the mangrove species could be an important tool for the forest managers to make decisions on future mangrove planting and forest protection via tracking population changes over time.

In this study, we use tree counts and environmental data collected from a network of 110 permanent sample plots distributed across the entire Sundarbans to generate spatially explicit baseline information on the distribution and habitat preferences of the four most abundant mangrove species i.e. *Heritiera fomes, Excoecaria agallocha, Ceriops decandra* and *Xylocarpus mekongensis*. We identify the key environmental variables related to their spatial distribution and generated species-specific spatial density maps using both geostatistical and regression approaches. We then demonstrated the potential applications of these habitat insights and spatial maps for future forest studies, spatial conservation planning and biodiversity protection and monitoring programs.

## Materials and methods

### Study system

**Figure 1:**
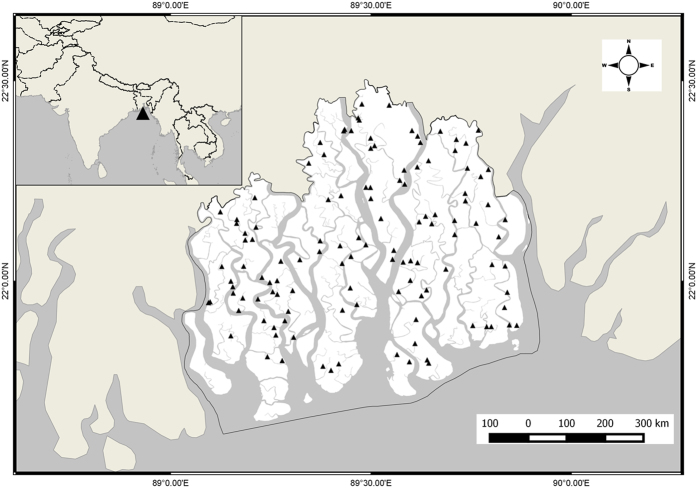
Sampling sites (triangles) in the Sundarbans, Bangladesh. The map was created using the software QGIS (version 2.10.1, URL: http://www.qgis.org/en/site/).

The Bangladesh Sundarbans (21°30′–22°30′N, 89° 00′–89°55′E) is part of the world’s largest river delta at the Ganges-Brahmaputra estuary (Fig. 1). Geologically, the Sundarbans is of recent origin (about 7000 years old) and was formed through the silt deposition by the Ganges-Brahmaputra river system^19^. The young, slightly calcareous soil is finely textured, poorly drained, rich in alkali metal contents, and with no distinct horizon in the sediment deposits^7^. Of its total area (6017 km^2^), about 69% is land and the rest comprises rivers, small streams and canals^9^. A major portion of this forest is washed by the tide twice a day and the water level is related to the combined effects of the seawater tides in the Bay of Bengal and freshwater input from the Ganges. During the monsoon (June–September), freshwater flow increases and during the dry season (October to May), fresh water flow sharply drops because of the reduced water influx from the Ganges. The climate is humid, maritime and tropical. Mean annual precipitation is 1700 mm (range: 1474 to 2265 mm); and mean maximum annual temperature is between 29.4°–31.3 °C (range: 9.3° to 40 °C)^20^.

### Tree surveys

The Bangladesh Forest Department (BFD) established a network of 120 permanent sample plots (PSPs) in the Sundarbans in 1986 for monitoring biodiversity and forest stock (Fig. 1). Of these, 110 PSPs (120 × 20 m (0.2. ha), divided into 5 20 × 20 m subquadrats) were positioned to represent the ecological zones (i.e. freshwater, moderately saline, and saltwater zones), and the forest types^12^. The remaining relatively smaller sized 10 PSPs (20 × 10 m) were established to monitor the ground vegetation mainly in the south-western Sundarbans, and were not considered in this study. The BFD tagged with a unique tree number and measured every tree with stem diameter ≥4.6 cm (recorded at 1.3 m from the ground). The height of each tree was also recorded. In this study, we used BFD’s last tree data (2008–2013) for 91 PSPs, and the tree data we collected (January – June 2014) for 19 PSPs.

### Environmental data

We collected environmental data from all 110 PSPs during January – June 2014. We adopted a soil sampling design (Supplementary Figure S1), collected 9 soil samples (to account for the within plot variation in soil parameters) from each PSP to a depth of 15 cm in polyethylene bags using a cylindrical soil core sampler of 5 cm in diameter for laboratory analysis. For soil texture analysis (percentage of sand, slit and clay), we used the hydrometer method^21^. We determined soil salinity (as electrical conductivity - EC) in a 1:5 distilled water:soil dilution^22^ using a conductivity meter (Extech 341350A-P Oyster). Soil pH and oxidation reduction potential (ORP) were measured in the field using digital soil pH and ORP (Extech RE300 ExStik) meters. Soil ammonium concentration (NH_4_) was determined following the Kjeldahl method^23^. We measured total phosphorus (P) using the molybdovanadate method and a 721-spectrophotometer. Soil potassium (K), magnesium (Mg), iron (Fe), zinc (Zn), copper (Cu), and sulfide concentrations were measured using an atomic absorption spectrophotometer (AA-7000). We analyzed each soil sample and then averaged (9 samples) the results. Five elevation (above-average sea level) readings for each PSP were extracted from the digital elevation model with accuracy (i.e. accuracy at pixel level) ±1 m for the Sundarbans region available in BFD^24^. We then averaged these 5 readings to minimize the error related to the digital elevation model. A proportional distance from the river-sea interface was used to calculate and classify “upriver position” (henceforth, URP) of each PSP^25^. Here ‘downstream’ represents the lower third (0–33% upriver from sea) of the estuarine system, ‘intermediate’ represents the middle third (34–66% upriver from sea), and ‘upstream’ represents the upper third (67–100% upriver from sea). This scheme is useful to understand each mangrove’s habitat preference along the downstream (seawater dominated river system) - upstream (freshwater dominated river system) gradient.

### Covariate selection for mangrove HSMs

To construct a biologically informative covariate set for our HSMs, we followed the conceptual framework developed for mangroves^26^. This comprises three broad categories of variables (i.e. resources, regulators and hydroperiod) that are believed to control mangrove forest structure and function^27^,^28^,^29^. Resources (i.e. nutrients) are depleted by mangrove trees and their availability is linked to tree productivity and indirect competition among individual trees. Here, we used soil NH_4_, P, K, Mg, Fe, and Zn based upon the detailed explanation of nutrient requirements of mangroves available in the mangrove literature^28^. Regulators are non-resource variables that influence mangrove growth. We employed soil salinity as our main regulator. Hydroperiod (the duration, frequency and depth of inundation) is recognized as an important determinant of mangrove distribution^29^. Sundarbans PSP-based hydroperiod data are available, so we used PSP elevation as a proxy that reflects the likely variation in hydroperiod across the area. We also included URP of each PSP as a predictor to account for the influence of the river systems on each mangrove’s distributions along the downstream-upstream gradient.

The relative abundance of one mangrove species might influence the abundance of another via biotic interactions i.e. competition or facilitation^30^ and each individual tree interacts with the trees (both conspecifics and heterospecifics) that are in its neighbourhood through multiple concurrent interactions^31^. Given the super-dominance of *E. agallocha* and *H. fomes* (see Tree surveys section) and tree structural complexities (i.e. multiple stems in *C. decandra*, large below ground biomass for modified root systems in *H. fomes, C. decandra*, and *X. mekongensis*) in the ecosystem which might have increased tree measurement (i.e. diameter, height) errors, we initially considered two alternative measures of abundance: 1) density of all stems (including stems on multiple stemmed individual) for each plot, and 2) total basal area (including stems on multiple stemmed individual) for each plot as biotic variables. HSMs of species with basal area as a covariate had lower explanatory and predictive powers, compared to models with density of all stems. Therefore, we selected density of all stems for each plot (henceforth, DAS) as the biotic variable. We acknowledge that the salinity-stressed western and southern habitats of the Sundarbans have many small diameter (with stunted growth) *E. agallocha* and *H. fomes* trees^32^ that may not compete. Disentangling biotic influences from abiotic effects in structuring ecological communities and regulating single species distributions is still an open problem^33^. However, the inclusion of stem densities as a simple proxy of biotic interaction is known to enhance the explanatory and predictive power of HSMs for other forest systems^34^,^33^.

The Sundarbans has a long exploitation history^7^. The government banned tree harvesting in 1989^35^. About 3.5 million people depend on Sundarbans resources (e.g. fish, non-timber forest products, honey) for their livelihoods and illegal tree harvesting is common^19^. Therefore, we included historical harvesting (henceforth, HH) as a covariate in our models because of its potential influence on present tree densities in the PSPs. HH represents the number of illegally harvested trees (detected by counting stumps) in each PSP from the first census (1986) to the last census (2014).

We checked for multi-collinearity in our set of candidate covariates by employing Variance Inflation Factors (VIF) through a stepwise model selection procedure. We used the *vifstep* function of ‘usdm’ package^36^ in R 3.2.2^37^, which first calculates VIF for all covariates, then eliminates the one with highest VIF that exceeds the threshold of 2.5 and repeats the procedure until no covariate with VIF >2.5 remains. This led to the removal of Zn from our covariate set (Supplementary Table S1).

### Habitat modelling

We used generalized additive models^38^ (GAMs) with a Poisson likelihood and a log-link because of their ability to handle complex, non-monotonic relationships between the response and the predictor variables^39^. Moreover, by using non-parametric smoothing functions, GAMs can often construct biologically insightful relationships between response and covariates without a-priori hypotheses^17^. Smoothed responses used cubic basis splines implemented within the ‘mgcv’ package^40^ in R.

We used the ‘dredge’ function in the ‘MuMIn’ package^41^ to fit all possible candidate models with all possible combinations of covariates and ranked the resulting models by the Akaike Information Criterion (AIC)^42^. We then obtained the relative support for each model by calculating the ∆AIC (difference between the AIC value for the best model and the AIC value for each of the other models). Kullback–Leibler information loss is minimal between models with ∆AIC ≤ 2^42^. We therefore used the ‘∆AIC ≤ 2’ criterion to select our confidence set of models for each mangrove species. We then calculated Akaike weights (AICw) to examine relative support for each model in the confidence set. AICw values range from 0 to 1 and the sum of all AICw across the confidence set is 1. When there was only one model with ∆AIC ≤ 2, it was unambiguous that it outperformed all possible candidate models. When there were multiple competing models, we used AIC-weighted model averaging on the parameter estimates of these models to reduce model selection uncertainty and bias. These averaged parameter estimates were used to predict the abundance of the mangroves species across the entire Sundarbans. Relative Importance (RI) of each covariate was identified by summing the AICw of the models in which the covariate was included. RI values range between 0 and 1, where 0 indicates that the target covariate is never included in the competing models, 1 indicates inclusion of the covariate in all the competing models. We ranked the covariates based on their RI values. Residual diagnostic plots for the best GAMs did not indicate violations of the Poisson dispersion assumption.

We measured goodness-of-fit of the models using the *R*^2^ (coefficient of determination) statistic between the observed and estimated abundance values. For validation purposes, we partitioned our dataset into calibration and validation subsets. Our calibration dataset included 88 PSPs (80% of the full data) and the validation data set included 22 PSPs (20%). The validation dataset was randomly chosen to cover the whole region and was used to examine the predictive power of the fitted models via the *R*^2^ statistic applied to the model’s predictions for the validation data. We also mapped the actual and predicted abundances of both calibration and validation set to check for any spatial patterns of prediction errors.

### Spatial mapping

We mapped densities of the mangrove species over the entire Sundarbans using two different approaches: 1) direct interpolation of plot-level raw abundance using geostatistical methods, and 2) habitat-based predictions from our HSMs. Both of these approaches were used because environmental data collection is demanding whereas tree abundance measurements are taken regularly at the PSPs, and it is useful to know how close the predictions of the habitat model were compared to simple interpolation methods. To directly interpolate individual mangrove species abundances, we used Ordinary kriging (OK), a widely used interpolation technique.

The model-averaged predictions from our confidence set of GAMs were used to develop the mangrove habitat maps based on interpolated covariate surfaces. The size of each grid-cell of the interpolated surface was 625 m^2^ (25 m × 25 m). Covariate surfaces for generating predictions from the habitat model were constructed by OK using the ‘gstat’ package^43^ in R. A protected area network comprising three Wildlife Sanctuaries (WS) – East WS, West WS, and South WS has been operational since 1960. We superimposed the protected area network on the habitat maps to assess the existing network’s ability to support density hotspots of the mangrove species. We compared the predictive abilities of the direct and habitat-based approaches using the normalized root mean square error (NRMSE) statistic derived from the leave-one-out cross-validation procedure. For normalization, the root mean square error statistic was divided by the range of the actual species abundances. Both habitat-based and direct predictions of the mangrove tree abundances were mapped using the ‘raster’ package^44^ in R. We further mapped the prediction discrepancy between these two approaches, to look for any spatial patterning in the prediction errors.

## Results

### Tree surveys

A single survey of each of the 110 PSP’s carried out between 2008–2014 gave a total of 49409 trees of 19 species from 13 families and 19 genera (Table 1). The most abundant mangrove was *E. agallocha* (59.69% of total trees), followed by *H. fomes* (30.89%), *C. decandra* (6.12%), and *X. mekongensis* (0.82%). The rest of the 15 species were extremely rare comprising only 2.49% of the total count.

### Habitat models

**Figure 2:**
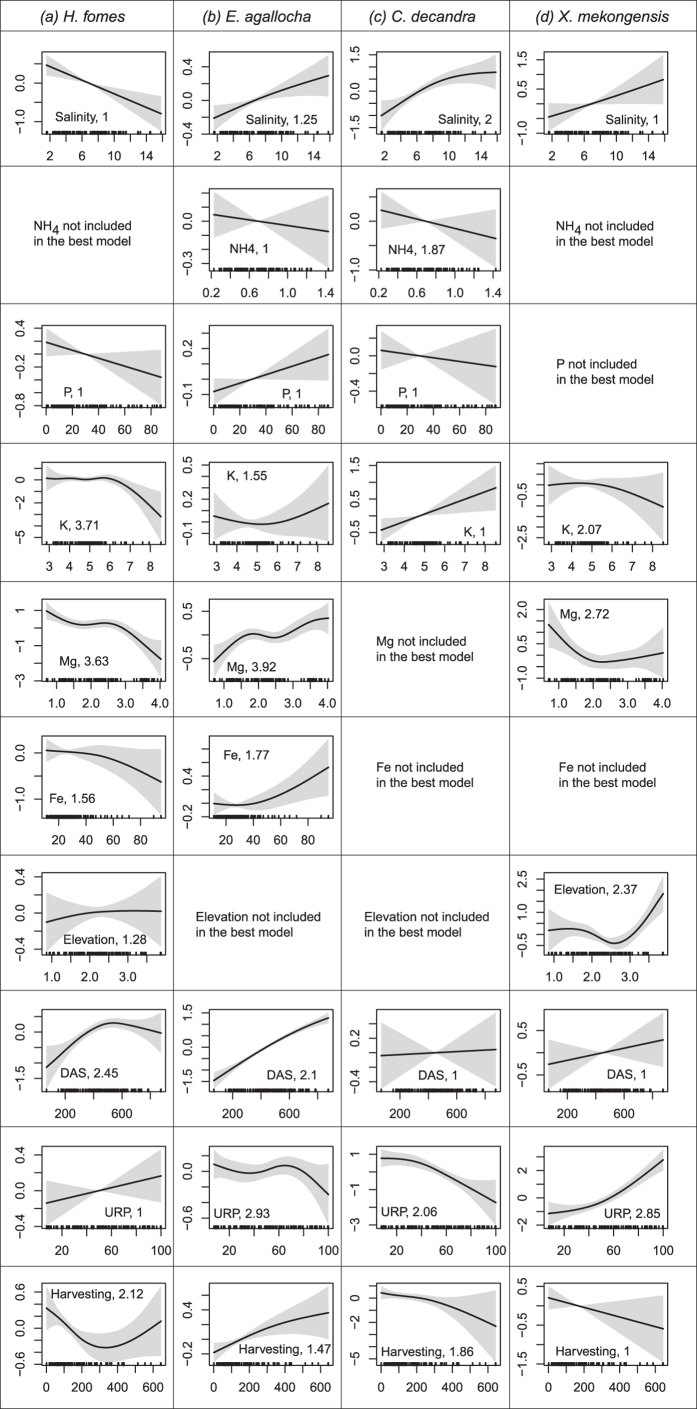
Effects of covariates inferred from our best GAMs fitted to the abundances of the four prominent mangrove species in the Sundarbans. The solid line in each plot is the estimated spline function (on the scale of the linear predictor) and shaded areas represent the 95% confidence intervals. Estimated degrees of freedom are provided for each smoother following the covariate names. Zero on the y-axis indicates no effect of the covariate on mangrove abundances (given that the other covariates are included in the model). Covariate units: soil salinity = dS m^−1^, elevation = m (above average-sea), NH_4_ = gm Kg^−1^, P = mg Kg^−1^, K = gm Kg^−1^), Mg = gm Kg^−1^, Fe = gm Kg^−1^, URP = % upriver, DAS = density of all stems for each plot, and historical harvesting (HH) = total number of harvested trees in each plot since 1986.

The most parsimonious GAMs for estimating species abundances explained the variability of *H. fomes* (68%), *E. agallocha* (84%), *C. decandra* (73%), and *X. mekongensis* (75%) (Table 2). Soil salinity, K, total density of individuals (DAS), upriver position (URP) and historical harvesting (HH) were included in the best GAMs of all species. Mg and Fe were included (RI = 1.00) in the best GAMs for *H. fomes, E. agallocha* and *X. mekongensis*, with P (RI = 1.00) for *H. fomes, E. agallocha*, and *C. decandra*, and also elevation (RI = 1.00) for *H. fomes* and *X. mekongensis*. The partial response plots of the best GAM (Fig. 2) indicated that *H. fomes* abundance decreased with increasing soil salinity (>7 dS m^−1^). In contrast, increasing salinity was associated with increasing abundances of *E. agallocha* (>7 dS m^−1^), *C. decandra* (>6.2 dS m^−1^), and *X. mekongensis* (>7 dS m^−1^).

The response of the mangrove species varied for different nutrients. High abundance of *H. fomes* was associated with low chemical concentrations of P (<30 mg Kg^−1^), K (<6 gm Kg^−1^), Mg (<2.75 gm Kg^−1^) and Fe (<30 gm Kg^−1^). In contrast, high *E. agallocha* abundance was associated with relatively high concentrations of P (>30 mg Kg^−1^), K (>6 gm Kg^−1^), Mg (>2.75 gm Kg^−1^), and Fe (>30 gm Kg^−1^), and low concentrations of NH_4_ (<0.70 gm Kg^−1^). High *C. decandra* abundance was related to high K (>5 gm Kg^−1^) and low NH_4_ (<0.70 gm Kg^−1^) and P (<30 mg Kg^−1^) concentrations. High *X. mekongensis* abundance was related to low K (<5 gm Kg^−1^) and Mg (<1.60 gm Kg^−1^).

*H. fomes* and *X. mekongensis* showed preferences for elevated sites. *H. fomes* abundances showed a decreasing trend after a certain value of the biotic variable DAS (>500 trees/0.2 ha), while *E. agallocha, C. decandra* and *X. mekongensis* showed positive responses to increasing DAS. With increasing URP the abundances of *E. agallocha* (URP > 65%) and *C. decandra* (URP > 50%) sharply decreased, indicating their high preference for down- and mid-stream habitats. In contrast, *H. fomes* and *X. mekongensis* abundances increased with increasing URP (>50%, indicating their preference for upstream habitats). High historical harvesting of trees was related to low abundances of *H. fomes, C. decandra* and *X. mekongensis*. In contrast, *E. agallocha* had high abundance in the sites that experienced high historical harvesting.

The predictive abilities of the GAMs (fitted to the calibration data and applied to the validation data) were *R*^*2*^ = 0.75 for *H. fomes, R*^*2*^ = 0.78 for *E. agallocha*, and *R*^*2*^ = 0.51 for *C. decandra*. The predictive ability of the *X. mekongensis* GAMs was somewhat lower (*R*^*2*^ = 0.24) than for the other mangrove species (possibly due to high densities in few upstream areas and overall low abundance in the entire region). When GAMs were used to estimate mangrove abundances for all 110 PSPs, we observed a strong association (*H. fomes, R*^*2*^ = 0.67; *E. agallocha, R*^*2*^ = 0.83; *C. decandra, R*^*2*^ = 0.65; *X. mekongensis, R*^*2*^ = 0.84) between the actual and estimated abundances. Spatial maps of the actual and estimated abundances of the mangroves (both calibration and validation datasets) looked similar and the residuals did not show spatial clustering (Supplementary Figures S2 & S3).

### Spatial distribution maps

**Figure 3:**
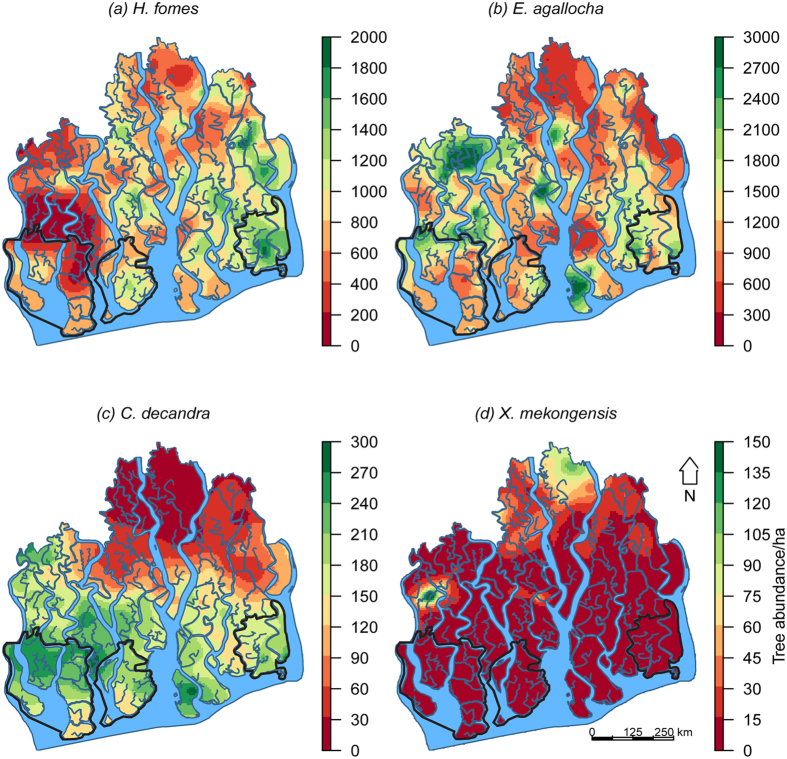
Spatial density ha^−1^ of the mangrove species in the Sundarbans based on habitat-based models (GAMs). Areas inside the bold black lines represent the three protected areas. The maps were created using ‘raster’ package (version 2.4–20) in software R (version 3.2.2, URL: https://cran.r-project.org/web/packages/raster/index.html).

**Figure 4:**
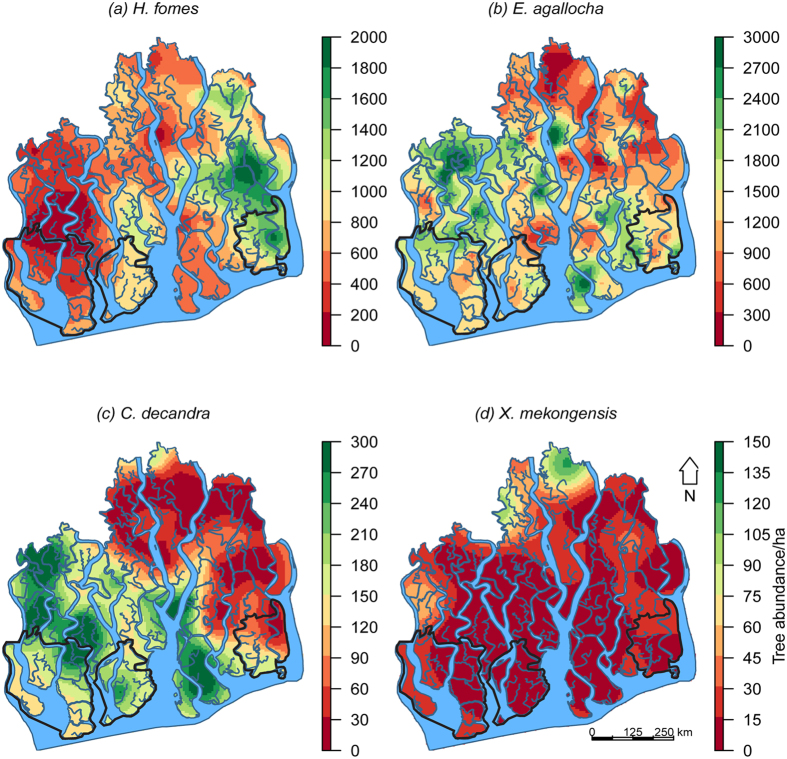
Spatial density ha-1 of the mangrove species in the Sundarbans based on geostatistical technique (OK). Areas inside the bold black lines represent the three protected areas. The maps were created using ‘raster’ package (version 2.4–20) in software R (version 3.2.2, URL: https://cran.r-project.org/web/packages/raster/index.html).

Habitat maps of the mangrove species based on GAMs, and direct interpolation (kriging raw abundances) are presented in Fig. 3 and 4. GAMs for *E. agallocha* had better predictive accuracies than direct interpolation (Supplementary Table S2). For *H. fomes, C. decandra* and *X. mekongensis*, both of these methods had almost identical predictive performances. Habitat mapping uncertainties related to these methods are presented in Supplementary Figure S4.

Overall, these maps indicate that the *H. fomes* density hotspots were confined to the eastern Sundarbans. *E. agallocha* density was highest in the north-western region, intermediate in the southern and eastern regions, and lowest in the northern and north-eastern regions. *C. decandra* density was highest in the western and southern regions, intermediate in the central region, and lowest in the northern and north-eastern regions. *X. mekongensis* density was highest in some specific areas in the northern (Kalabogi and Koyra) and north-western (Koikhali) regions. All the three protected areas – East WS, West WS, and South WS are distributed in the downstream areas (adjacent to the Bay of Bengal) (Fig. 3), and do not support the density hotspots for any of these mangrove species.

## Discussion

Our study is the first to quantify mangrove habitat suitability and to determine the key drivers regulating spatial distributions of the mangrove species in the Sundarbans world heritage ecosystem. The high explanatory and predictive power of these HSMs confirm their potential usefulness for constructing regional habitat maps to aid mangrove conservation initiatives. In addition, their ability to reveal mangroves’ responses to environmental and biotic predictors provides novel insights into the underlying ecology of these poorly understood but threatened mangrove species.

Extreme salt stress impedes growth and development of many mangroves^45^ and the structural development of mangrove forests tends to be limited by high levels of salinity^46^. In the Sundarbans, the response of the mangrove species varies steeply across the salinity gradient (Fig. 2). *H. fomes* shows a clear negative response (high density in the less saline and freshwater rich eastern habitats) and the three other mangroves (*E. agallocha, C. decandra* and *X. mekongensis*) show clear positive responses to increasing soil salinity with high densities in hyper-saline western and southern habitats (Fig. 3 & 4).

Our results indicate that the magnitude of response to nutrients varies across mangrove species. *E. agallocha* and *C. decandra* are able to grow abundantly in the NH_4_-poor habitats. Limited soil P is a key constraint for forest productivity in tropical ecosystems^47^. *E. agallocha* prefers relatively P-rich habitats (>30 mg Kg^−1^). In contrast, *H. fomes* grows abundantly in the P limited sites (<30 mg Kg^−1^). Soil K is considered as the key macro-nutrient that can modulate salinity-induced drought stress by improving the water uptake and retention capacity of plants^48^. Relatively higher densities of *E. agallocha*, and *C. decandra* in the highly saline and relatively K-rich habitats (north-western and southern Sundarbans) indicate that these species might have developed strategies for efficient utilization of K in salinity stressed habitats. Fe and Mg are required for successful mangrove growth because of their roles in metabolic and physiological processes^49^. *E. agallocha* clearly prefers Fe-rich habitats, whilst *H. fomes* prefers Fe-poor habitats. The Mg preference range of *E. agallocha* (>2.75 gm Kg^−1^) is somewhat higher than that of *H. fomes* (<2.75 gm Kg^−1^) and *X. mekongensis* (<1.60 gm Kg^−1^). This disparity may be related to mechanisms (e.g. the distribution and chemical properties of the source rock material, the weathering process, and salinity levels) that control availability of Mg to plants^50^. It is worth remembering that in this study the fitted response curves for each mangrove species only just describes how its densities are correlated with multiple predictors within their observed environmental ranges. Since these predictors include proxies for competition, these curves do not necessarily reveal the physiological limits (i.e. the fundamental niche) of the mangroves.

Although the Sundarbans is a deltaic swamp with narrow elevation gradient (0.50 m–4.0 m above mean sea level), it is characterized by diverse elevation values. The western zone is more elevated than the eastern zone because of tectonic activity and higher sediment deposition. This variation may be responsible for variable inundation levels in the mangrove habitats with consequent differences in soil salinity and available nutrients, and may ultimately have forced the mangrove trees to be distributed in distinct zones^51^. This hypothesis was tested^8^ using randomization tests and data from 11 sampling stations in the Sundarbans, and the researchers in that study concluded that the mangroves of the Sundarbans show no distributional patterns along the elevation gradient (i.e. absence of zonation). In contrast, our results show that *H. fomes* (>2.00 m) and *X. mekongensis* (>2.75 m) show clear preference for elevated sites (Fig. 2). We were able to reveal these patterns because of our larger sample size of 110 PSPs distributed over the entire region, and our multivariate and nonlinear modeling methodology.

DAS i.e. density of all stems for each plot (with maximum RI (~1) scores) was retained in the species best GAMs, indicating the strength of adding biotic variables in environmental data driven HSMs. *H. fomes* abundance tends to fall when the DAS value is >500 trees/0.2 ha, indicating the super dominance of generalists (i.e. *E. agallocha* – shows positive linear response to DAS) and disturbance specialists (i.e. *C. decandra*). The negative association of *H. fomes* with *E. agallocha* and *C. decandra* was observed in a previous study in the Sundarbans^8^. Conversely, the abundance of *X. mekongensis* is higher in the highly populated habitats. Indeed, these are the northern *X. mekongensis* hotspots (Fig. 3) where *X. mekongensis* is positively associated with *H. fomes* and *Bruguiera gymnorrhiza*. Although our correlative inferences might not necessarily reflect the causal mechanisms of biotic interactions (competition or facilitation) on species distributions, they do help improve explanatory and predictive power of HSMs and form the basis for more mechanistic studies.

URP, representing the downstream-upstream gradient, was retained in all of the selected GAMs with maximum RI score (~1), indicating the influence of river systems on the spatial distribution of mangroves. The river system covers about 1700 km^2^ (with maximum river width of 10 km) and continually change channels. Erosion and compensatory accretion are common along the river banks. The freshwater supply from these rivers mainly control the amount of alluvium deposit in the forest floor, which in turn regulate the availability of plant nutrients^7^. The negative response of *E. agallocha* and *C. decandra* abundances to increasing URP indicate their preference for habitats distributed between the downstream to intermediate positions (0 – 66% upriver from the sea – Bay of Bengal). Conversely, *H. fomes* and *X. mekongensis’s* clear positive response to URP (>50%) would support a characterization of these species as upland specialists. These disparities in mangrove habitat preferences along the downstream-upstream gradient may be related to the change in regional hydrology since the construction of the Farakka dam (1974) on the Ganges in India which has silted up most of its southbound distributaries heading towards the Sundarbans’ river system. As a result, the carrying capacities of the major river (e.g. Sibsa and Posur) systems have radically changed with about 60% reduction in the freshwater flow^9^.

*H. fomes* is now facing extinction in the Indian Sundarbans and Myanmar^52^. The Bangladesh Sundarbans now supports the sole remaining viable population of this globally endangered mangrove^19^. Our *H. fomes* habitat map (Fig. 3) indicates that the eastern region of the Sundarbans supports the highest *H. fomes* populations, the central and northern regions support intermediate densities, and the mangrove is almost absent in the western region. This may indicate historical range contraction of the species even in the Bangladesh Sundarbans as palynological evidence suggests its past dominance in the western region^20^. The sharp negative response of *H. fomes* to increasing intensity of historical tree harvesting (Fig. 2) indicates that this has been one of the main target species for illegal harvesting. In fact, *H. fomes* stem density has declined by 50% (1960s–1990s) all over the Sundarbans because of habitat degradation and mass exploitation^12^. *H. fomes* prefers freshwater dominated habitats and shows a negative response to increased soil salinity. Therefore, the highest abundances in the eastern region may be related to its proximity to the freshwater dominated Baleshwar River. However, the freshwater supply to the eastern zone has been decreasing because of heavy siltation in the internal channels^9^. Our findings lead us to conclude that further harvesting and decreases in freshwater supply (i.e. increased salinity) could push this species over the brink of extinction.

*E. agallocha* habitat maps indicate this species’ wide distribution across the entire Sundarbans, except the upstream-dominated northern region. Contrary to *H. fomes, E. agallocha* is a salt tolerant fast growing and reproducing species with high ability to colonize open and degraded habitats^53^. *E. agallocha* abundances increased in the sites with high historical harvesting intensity (Fig. 2). Tropical cyclones and tree mortality have created large forest gaps in the Sundarbans and the amount of open areas has been increasing by 0.05% each year^19^. Hence, we assume that these conditions may favour *E. agallocha* to increase its density and expand its range even to the upstream dominated northern region.

*C. decandra* hotspots are now distributed in the south and south-western zones (Fig. 3*). C. decandra* and other dwarf species have been replacing about 0.4% of the forest area every year^20^. Intermediate *C. decandra* densities in the central and south-eastern regions provide clear indication of its landward range expansion. Interestingly, although *C. decandra* belongs to the ‘Near Threatened’ status globally, its populations seem to be increasing and the species may be expanding its landward range.

High-density populations of *X. mekongensis* are restricted to few specific areas of the northern (Kalabogi and Koyra) and north-western (Koikhali) regions (Fig. 3). The distribution of the species is patchy in the rest of the ecosystem. *X. mekongensis* abundances show sharp negative response to increasing historical harvesting intensity (Fig. 2). This has been the target species for illegal felling since colonial regime because of its high timber price in the black market^54^. At present, most of the *X. mekongensis* trees (64%) are infected by the heart root disease^55^. Hence, *X. mekongensis* is under severe pressure in the Sundarbans, and could be at higher risk of local extinction.

The existing protected area network (East, West, and South Wildlife Sanctuaries) does not include the hotspots of any of these threatened species (Fig. 3). Our habitat maps advocate the immediate protection of the remaining suitable habitats (hotspots) of *H. fomes* and *X. mekongensis*, the two species most at risk of global and local extinction. According to the Bangladesh Wildlife Preservation Order 1973 (amended in 1974) these sanctuaries were established to ensure completely undisturbed habitat for the protection of wildlife, vegetation, soil and water^14^. The capacity of these sanctuaries to conserve biodiversity with limited physical and technological resources, has been highly disputed^14^. Given the circumstances, a preventative approach involving the design of a new or extended network of protected areas with improved logistics support is a plausible option offering expediency and cost effectiveness over long term forest restoration projects^56^.

The usefulness of HSMs in guiding species habitat restoration, protection, and replanting projects is well documented. Although identifying the potential existence of environmental stressors should be the first step in reforestation and restoration planning, a limited understanding of mangroves habitat requirements has limited the success of such initiatives in many countries^13^. In the Sundarbans, past replanting campaigns (based on educated guesses) were also unsuccessful^14^. In this context, the regional HSMs of this study with detailed information on the mangroves’ habitat requirements, may guide the future restoration and mangrove planting initiatives of the Bangladesh Forest Department. The absence of a persistent soil seed bank of *H. fomes* and *X. mekongensis* in the Sundarbans has recently been identified^53^. Thus, we recommend mangrove planting in the forest gaps, to safeguard these habitats from invasive species^57^.

The Sundarbans has a history of extensive exploitation particularly during the 1980s^7^. The government enforced a full logging ban in 1989^35^. Despite such law enforcement, illegal felling of trees is common^19^. Our results also indicate the negative influence of historical harvesting on the populations of the threatened mangrove species. This exploitation is also directly linked with the habitat loss of many mangrove-dependent animals including the globally endangered Royal Bengal tiger^58^. Bangladesh has signed and ratified the World Heritage Convention, Ramsar Convention and the Convention on Biological Diversity^35^. The government of Bangladesh has recently developed the Biodiversity National Assessment and Program of Action 2020 to implement sufficient measures to halt further degradation of biological resources. Therefore, our mangrove distribution maps may guide these valuable protection and monitoring initiatives of the Bangladesh Forest Department to combat illegal logging through recording mangrove population changes or predicting changes and identifying areas (or species) that may be most affected by future harvesting and other human interventions (e.g. settlement and shrimp farming).

## Conclusions

This study is the first to make complete inventories in the PSP network established in the 1980s by the Bangladesh government for monitoring biodiversity and forest health, demonstrates the usefulness of habitat modelling as a tool in predicting mangrove abundances and provides novel insights into the underlying ecology of these poorly studied threatened species. The HSMs and complementary habitat maps provide spatially explicit information on the remaining habitats of the threatened mangrove species, and form the baseline for designing cost-effective field inventories, biodiversity assessment and monitoring programs. Most importantly, the Bangladesh Forest Department can readily use the distribution maps in their existing protection and monitoring initiatives designed to combat illegal logging in the Sundarbans. The relative performance of the direct interpolation-based species distribution maps against the habitat-based spatial density maps indicates their usefulness when environmental data are not available. We did not make HSMs for the remaining 15 mangrove species in our data due to their low prevalence. Future studies may usefully extend their sampling efforts beyond the existing PSP network to record these rare mangroves. The projected sea level rise along the Bangladesh coast, which is higher than the global rate, may alter the hydrology of the Sundarbans with subsequent changes in the salinity and nutrient levels in the mangroves’ habitats. Therefore, we recommend including hydroperiod as a predictor in future HSMs as these data become available.

## Additional Information

**How to cite this article**: Sarker, S. K. *et al*. Are we failing to protect threatened mangroves in the Sundarbans world heritage ecosystem? *Sci. Rep.* **6**, 21234; doi: 10.1038/srep21234 (2016).

## Supplementary Material

Supplementary Information

## Figures and Tables

**Table 1:** Taxonomy, global conservation status, and mean abundances of the mangrove species censused in the 110 permanent sample plots in the Bangladesh Sundarbans.

Latin name	Local name	Family	IUCN conservation status	Global population trend*
*Aegiceras corniculatum* (L.) Blanco	Khalshi	Myrsinaceae	LC	D
*Amoora cucullata* Roxb.	Amur	Meliaceae	NA†	NA
*Avicennia officinalis* L.	Baen	Avicenniaceae	LC	D
*Bruguiera gymnorhiza* (L.) Lam.	Kakra	Rhizophoraceae	LC	D
*Cerbera manghas* L.	Dagor	Apocynaceae	NA	NA
*Ceriops decandra* (Griffith) Ding Hou	Goran	Rhizophoraceae	NT	D
*Cynometra ramiflora* L.	Singra	Fabaceae	NA	NA
*Excoecaria agallocha* L.	Gewa	Euphorbiaceae	LC	D
*Excoecaria indica* (Willd.) Müll.Arg.	Batul	Euphorbiaceae	DD	D
*Heritiera fomes* Buch.-Ham.	Sundri	Malvaceae	EN	D
*Intsia bijuga* (Colebr.) Kuntze	Bhaela	Leguminosae	VU	D
*Lumnitzera racemosa* Willd.	Kirpa	Combretaceae	LC	D
*Pongamia pinnata* (L.) Pierre	Karanja	Leguminosae	LC	Stable
*Rhizophora mucronata* Lam.	Jhana	Rhizophoraceae	LC	D
*Sonneratia apetala* Buch.-Ham.	Keora	Lythraceae	LC	D
*Talipariti tiliaceum* (L.) Fryxell	Bhola	Malvaceae	NA	NA
*Tamarix indica* Willd.	Nona Jhao	Tamaricaceae	NA	NA
*Xylocarpus granatum* Koen.	Dhundal	Meliaceae	LC	D
*Xylocarpus mekongensis* Pierre	Passur	Meliaceae	LC	D

^*^IUCN global population trend,

^†^Not assessed for the IUCN Red List, LC = Least concern, DD = Data deficient, NT = Near threatened, VU = Vulnerable, EN = Endangered, D = Decreasing.

**Table 2:** Results of generalized additive models (GAMs) built for the four major mangrove species of the Bangladesh Sundarbans.

Species	Model rank	Salinity	ELE	NH_4_	P	K	Mg	Fe	URP	DAS	HH	∆AIC	∆AICw	Adj-R^2^	DE (%)
***H. fomes***	1	+	+	─	+	+	+	+	+	+	+	0.00	0.99	0.67	68
	RI	1.0	1.0	0.0	1.0	1.0	1.0	1.0	1.0	1.0	1.0				
															
	1	+	─	+	+	+	+	+	+	+	+	0.00	0.66	0.83	84
***E. agallocha***	2	+	+	─	+	+	+	+	+	+	+	1.39	0.33		
	RI	1.0	0.67	0.67	1.0	1.0	1.0	1.0	1.0	1.0	1.0				
***C. decandra***															
	1	+	─	+	+	+	─	─	+	+	+	0.00	0.46	0.65	73
	2	─	+	+	+	+	─	─	+	+	+	0.53	0.35		
	3	+	+	+	+	+	─	─	+	+	+	1.84	0.18		
***X. mekongensis***	RI	0.65	0.65	1.0	1.0	1.0	0.0	0.0	1.0	1.0	1.0				
															
	1	+	+	─	─	+	+	─	+	+	+	0.00	0.75	0.84	75
	RI	1.0	1.0	0.0	0.0	1.0	1.0	0.0	1.0	1.0	1.0				

DE = deviance explained, RI = relative variable importance in the model selection process. Covariates: soil salinity, elevation above average-sea level (ELE), soil NH_4_, total phosphorus (P), potassium (K), magnesium (Mg), iron (Fe), upriver position (URP), density of all stems for each plot (DAS) and historical harvesting (HH).
