# Supplementary material for: Are we failing to protect threatened mangroves in the Sundarbans world heritage ecosystem?
Source: Sci Rep. 2016 Feb 16;6:21234. doi: 10.1038/srep21234 (PMC4754640; doi:10.1038/srep21234)

Are we failing to protect threatened mangroves in the Sundarbans world heritage ecosystem?

Swapan K. Sarker[1,4,*], Richard Reeve[1], Jill Thompson[2], Nirmal K. Paul[3] and Jason Matthiopoulos[1]

[1] Institute of Biodiversity, Animal Health and Comparative Medicine, University of Glasgow, Glasgow G12 8QQ, United Kingdom

[2] Centre for Ecology & Hydrology, Bush Estate, Penicuik, Midlothian, EH26 0QB, United Kingdom

[3] Management Plan Division, Bangladesh Forest Department, Khulna - 9100, Bangladesh

[4] Department of Forestry and Environmental Science, Shahjalal University of Science & Technology, Bangladesh

[*] Corresponding author

Supplementary Tables and Figures

Supplementary Table S1. Stepwise VIF test outputs of the environmental covariates.

Covariates	VIF
Salinity	1.53
NH4	1.63
P	1.32
K	1.35
Mg	2.13
Fe	1.58
Zn	2.60
Elevation	1.09
URP	1.07

Supplementary Table S2. Comparison of predictive accuracy (through leave-one-out cross validation) between the habit-based models (GAMs) and Ordinary kriging (OK) based on normalized root mean square error (NRMSE) of the predicted species abundances versus the actual abundances. NRMSE is expressed here as a percentage, where lower values indicate less residual variance.

	GAMs	OK
	NRMSE (%)	
H. fomes	20	20
E. agallocha	14	23
C. decandra	26	21
X. mekongensis	15	16

Supplementary Figure S1. Soil sampling design. Total 9 soil samples (circles, 0 – 30 cm depth) were randomly (3 samples/subplot) collected in the ends and middle of the 20 x 20 subplots in each PSP.

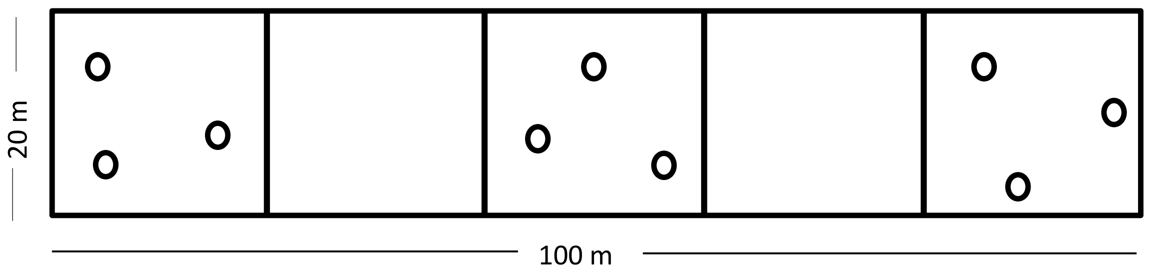

Supplementary Figure S2. Spatial distributions of the actual and estimated abundances of the four mangrove species, and uncertainties.

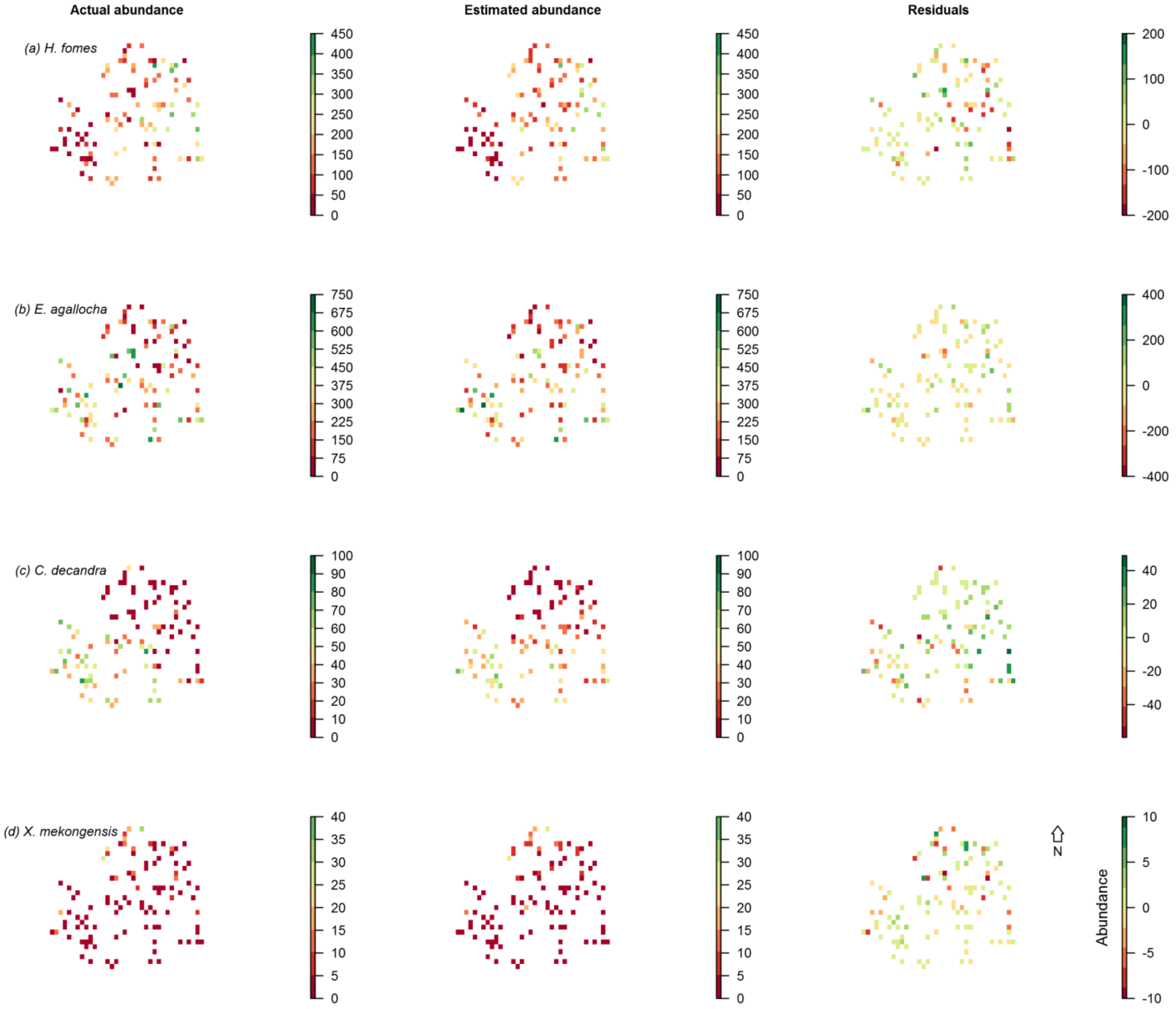

Supplementary Figure S3 Spatial distributions of the actual and estimated abundances of the four mangrove species, and uncertainties when the habitat models were applied to the validation data set.

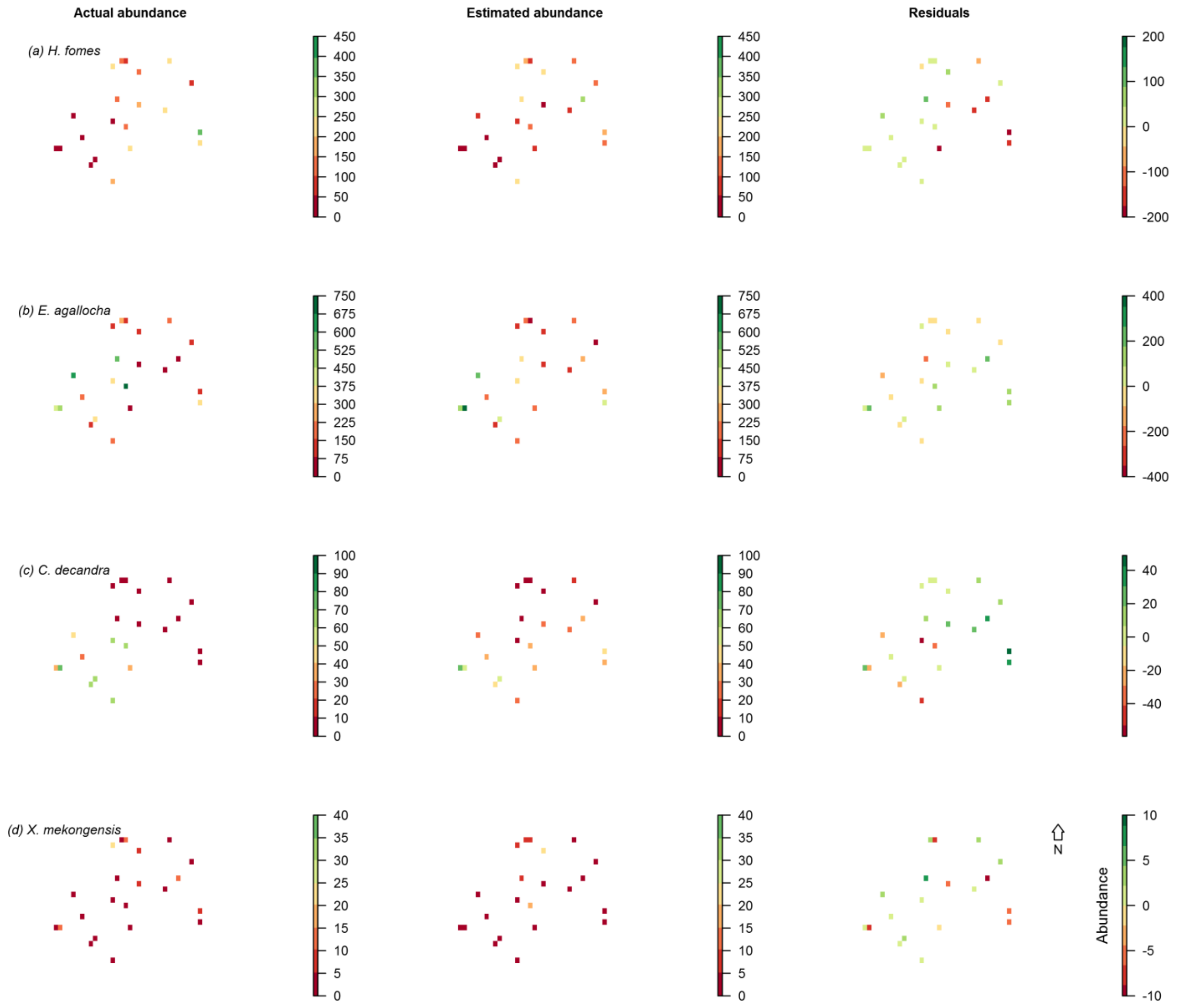

Supplementary Figure S4. Spatial distributions of the predicted abundance differences between the GAMs and Ordinary kriging for the four mangrove species. The maps were created using ‘raster’ package (version 2.4-20) in software R (version 3.2.2, URL: https://cran.r-project.org/web/packages/raster/index.html).

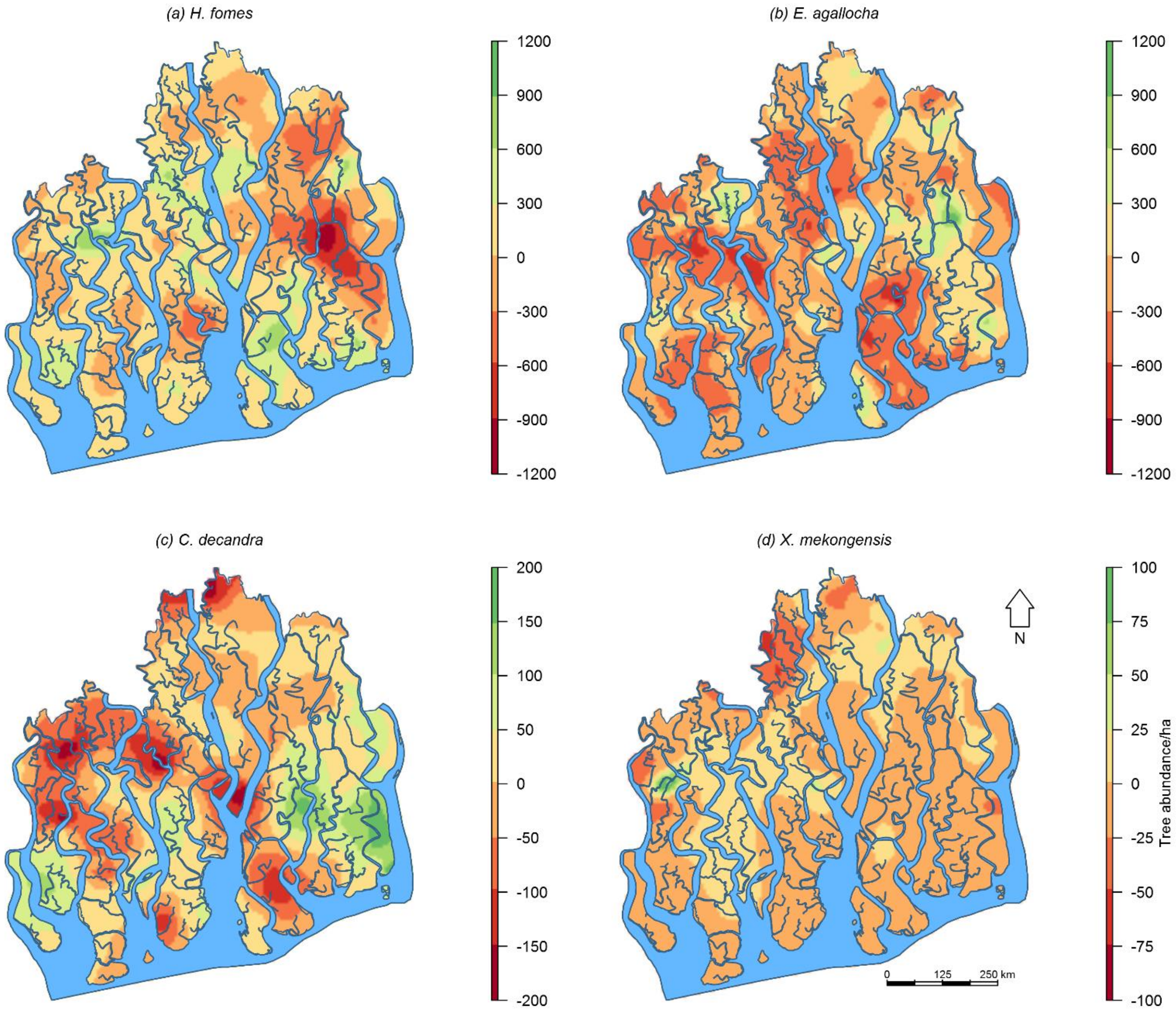

Supplement: Supplementary Information [file srep21234-s1.pdf]
